# Cultivated genome references for protein database construction and high-resolution taxonomic annotation in metaproteomics

**DOI:** 10.1128/spectrum.01755-24

**Published:** 2024-12-12

**Authors:** Xiaowei Gao, Hewei Liang, Tongyuan Hu, Yuanqiang Zou, Liang Xiao

**Affiliations:** 1 BGI Research, Shenzhen, China; 2 BGI Research, Wuhan, China; Lerner Research Institute, Cleveland, Ohio, USA

**Keywords:** metaproteomics, protein database, taxonomic analysis, functional analysis

## Abstract

Metaproteomics offers a profound understanding of the functional dynamics of the gut microbiome, which is crucial for personalized healthcare strategies. The selection of an appropriate database is a critical step for the identification of peptides and proteins, as well as for the provision of accurate taxonomic and functional annotations. The matched metagenomic-derived database is considered to be the best, but its limitations include the identification of low-abundance organisms and taxonomic resolution. Herein, we constructed a protein database (DBCGR2) based on Cultivated Genome Reference 2 (CGR2) and developed a complete peptide-centric analysis workflow for database searching and for the annotation of taxonomy and function. This workflow was subsequently appraised in comparison with metagenomics-derived databases for the analysis of metaproteomic data. Our findings suggested that the performance of DBCGR2 in identification was comparable with metagenomics-derived databases with improvement in identification rates of peptides from low-abundance species. The database searching results could be fully annotated using the pepTaxa taxonomic annotation approach developed in this study, and the taxonomic resolution was enhanced to strain level. Additionally, the results demonstrated that the sensitivity of functional annotation could be enhanced by employing DBCGR2. Overall, the DBCGR2 combined with pepTaxa can be considered an alternative for metaproteomic data analysis with superior analysis performances.

**IMPORTANCE:**

Mass spectrometry-based metaproteomics offers a profound understanding of the gut microbial taxonomy and functionality. The databases utilized in the analysis of metaproteomic data are crucial, as they determine the identification of proteins that can be recognized and linked to overall human health, in addition to the quality of taxonomic and functional annotation. Among the most effective approaches for constructing protein databases is the utilization of metagenomic sequencing to create matched databases. However, the database, derived from isolated genomes, has yet to undergo rigorous testing for their efficacy and accuracy in protein identification and taxonomic and functional annotation. Here, we constructed a protein database DBCGR2 derived from Cultivated Genome Reference 2 (CGR2) and a complete workflow for data analysis. We compared the performances of DBCGR2 and metagenomics-derived databases. Our results indicated that DBCGR2 can be regarded as an alternative to metagenomics-derived databases, which contribute to metaproteomic data analysis.

## INTRODUCTION

Metaproteomics, by enabling comprehensive profiling of proteins expressed by the gut microbiota, provides deep insights into the functional dynamics that are critical for understanding the gut ecosystem (1, 2). Such insights have significant implications for clinical interventions and personalized healthcare strategies, as they allow for the identification of target proteins involved in disease processes (3). For instance, proteolytic enzymes from *Bacteroides vulgatus* play a role in gastrointestinal diseases, and thus, their inhibition is seen as a promising therapeutic avenue (4–6). Research using metaproteomics has discovered that the microbial protein GroEL is overexpressed in the gut microbiota of individuals with type 2 diabetes, highlighting its potential as a novel therapeutic target for the disease (1). Moreover, complementing sequencing-based methodologies, metaproteomics offers a unique perspective on the microbial community structure by focusing on the protein biomass, which is a direct reflection of the functional state of the microbiota (7–9).

The canonical technique for metaproteomic analysis is a multi-step mass spectrometry (MS)-based shotgun bottom-up metaproteomics workflow beginning with protein extraction from the samples. This extraction is followed by enzymatic peptide digestion, and the peptides are analyzed using liquid chromatography tandem mass spectrometry. The analysis produces raw data, which are then subjected to database searching and bioinformatics interpretation including taxonomic and functional analysis. Database selection is crucial since it affects peptide and protein identification along with achieving accurate taxonomic and functional resolution and accuracy in the field of metaproteomics. Theoretically, the database should include all the protein sequences from the gut microbial community. In metaproteomic research, this becomes significantly more difficult when the composition of the gut microbiome remains unidentified at the strain level. The complexity of the gut microbiota and the variability between individuals add further challenges to the construction of databases.

Currently, the databases generated based on metagenomic data are the most commonly used (10, 11), which enable the identification of a diverse range of microbial organisms, including those that cannot be cultivated. The metagenomics-derived databases include sample-matched metagenomics-derived databases and comprehensive catalogs of reference genes in the human gut microbiome, such as the the Integrated Gene Catalog (IGC) database (12). Yet, the database construction strategy based on metagenomics has its limitations. It may not effectively capture organisms present in low abundance (13, 14). Some of the low-abundance taxa, which may neither be assembled into metagenome-assembled genomes nor pass quality filtering (15), are assumed to play an important role in human health (16, 17). Metagenomic approaches also suffer from incomplete genomic representation and require specialized expertise for data management. The short-read nature of metagenomic sequencing makes accurate species-level protein taxonomic assignments difficult, an issue that is exacerbated by the lack of taxonomic annotations within the datasets (14, 18). Additionally, databases constructed based on metagenomic data sets encounter challenges regarding consistency among various studies owing to the fluctuation in protein identifiers.

Conversely, organisms isolated and cultured from the gut enable the acquisition of complete and accurate genome information, which have advantages in taxonomic annotation of metaproteomic data. Our laboratory previously established a collection called CGR2, which includes 3,324 bacterial genomes isolated from healthy Chinese (19). CGR2 encompasses the majority of the most abundant organisms present in the gut microbiome and could increase the mapping rates by providing information from novel species (19). A previous study showed that a culture-based database construction strategy can accurately reflect the taxonomic composition of the gut microbiota (20). Another study showed that the extensive whole-genome databases can be a viable alternative in the identification of low-abundance taxa by achieving similar results with metagenomics-derived databases (15). Therefore, we hypothesized that a protein database constructed based on the CGR2 genomes could be utilized in metaproteomic data processing, which may increase peptides or protein identification rates from low-abundance taxa and enable high taxonomic resolution.

In this study, we used the culturomics-derived database construction strategy to generate a database (DBCGR2) from the information in CGR2 alone for metaproteomic data analysis. We accomplished a complete peptide-centric analysis workflow and evaluated the performances of DBCGR2 for identification rate and taxonomic and functional annotation. The results showed that DBCGR2 can be used in the whole metaproteomic data analysis. DBCGR2 can be considered an alternative to metagenomics-derived databases and can provide high taxonomic resolution and sensitive functional annotation.

## MATERIALS AND METHODS

### Construction of DBCGR2 protein database

**Fig 1:**
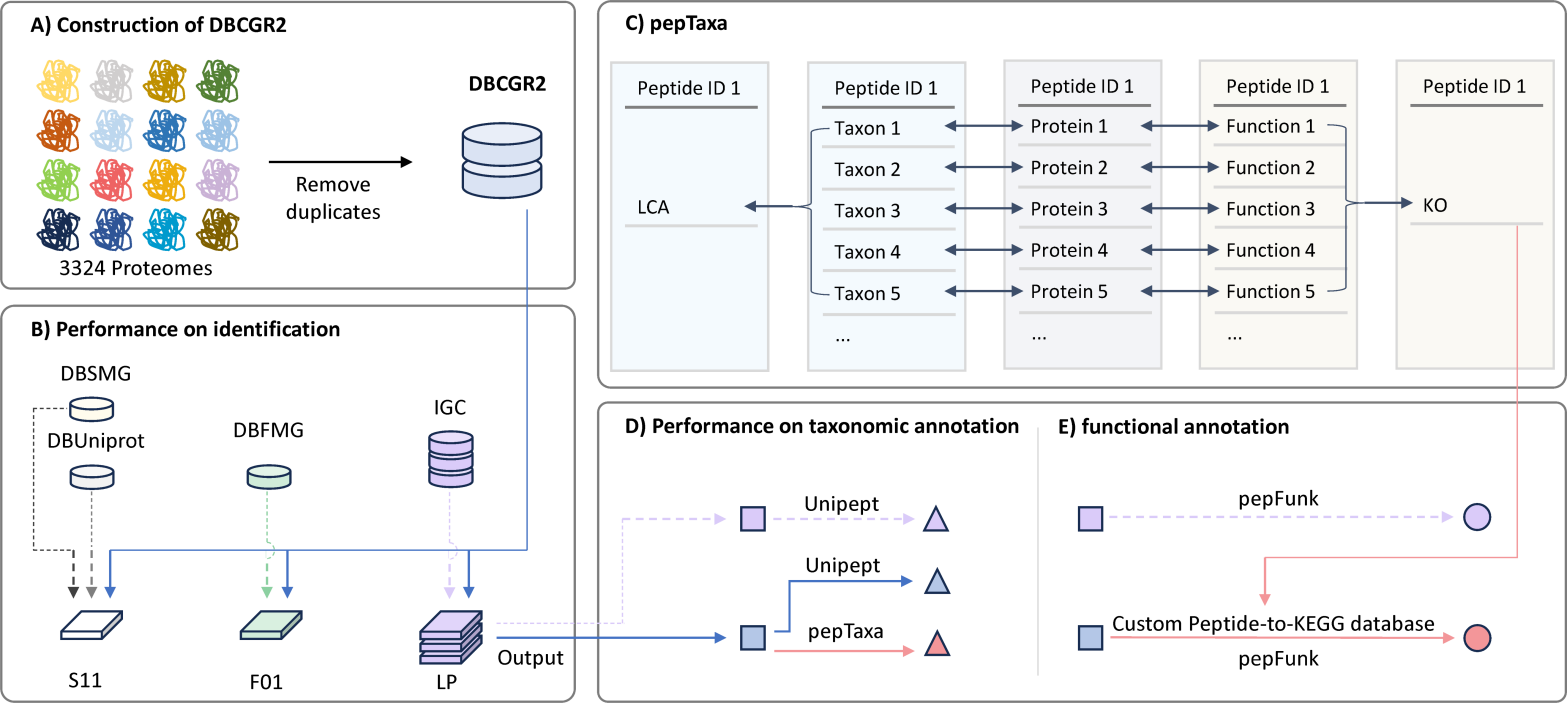
Workflow for evaluating the performance of DBCGR2 in identification and taxonomic annotation.

The CGR2 collection contains 3,324 reference genomes and corresponding proteomes, isolated from 454 healthy Chinese donors (19, 21) (Fig. 1A). The generation of corresponding proteomes based on genomes and taxonomic and functional annotation [Kyoto Encyclopedia of Genes and Genomes (KEGG)] was described in a previous study (19). To construct the DBCGR2 protein database, 3,324 proteomes were combined. Then duplicate sequences with 100% identity of full length were removed using CD-HIT (v4.6.8) (22). The final DBCGR2 protein database contains 4,043,864 non-redundant protein sequences (1.51 GB).

### Data sets used for evaluation

To assess the application scenarios of DBCGR2, we evaluated it on two types of samples: samples with known bacterial composition and fecal samples. We used the data sets of samples with known bacterial composition and fecal samples from Critical Assessment of MetaProteome Investigation (CAMPI), which were used for other metaproteomic workflow evaluations (23, 24). In the CAMPI project, matched-metagenomics databases were available for database searching comparison. We further adopted a data set collected from a Chinese cohort to study the performance of DBCGR2 on a specific population, where a gene catalog database IGC was used for database searching.

The data set of a synthetic microbial community, named SIHUMIx, was obtained from the CAMPI project (25). The SIHUMIx sample contains eight species: *Anaerostipes caccae* DSMZ 14662, C*lostridium butyricum* DSMZ 10702, *Erysipelatoclostridium ramosum* DSMZ 1402, *Lactobacillus plantarum* DSMZ 20174, *Bifidobacterium longum* NCC 2705, *Bacteroides thetaiotaomicron* DSM 2079, *Blautia producta* DSMZ 2950, and *Escherichia coli* MG1655. In this study, we used the metaproteomic raw data of sample S11. The corresponding protein database derived from metagenomic sequencing data from S11 was downloaded and termed DBSMG in this study. The DBSMG contains 19,319 protein sequences (6.1 MB). A DBUniprot protein database was also downloaded from the CAMPI, which was constructed by the combination of proteomes of strains in SIHUMIx, which were downloaded from UniProt. The DBUniprot contains 29,557 protein sequences (13.2 MB).

For the analysis of the fecal sample from CAMPI project (25), we used the sample F01 data set. The fecal sample was collected from a healthy adult. The corresponding protein database derived from metagenomic and metatranscriptomic sequencing data was downloaded and named DBFMG. The DBFMG contains 441,558 protein sequences (114.4 MB).

In the “Lunar Palace 365” (LP) project, eight crews resided in the closed manned Bioregenerative Life Support System facility (26). A total of 90 fecal samples were taken at different time points within a year. The LP data set was used as a time-resolved fecal metaproteomic data set from a Chinese cohort. The protein database IGC used in the original study was constructed as a gene catalog protein database using multiple metagenomic data sets from human gut (12), containing 9,879,896 protein sequences (2.6 GB).

### Metaproteomic raw data processing

Raw data were processed by MaxQuant (version 2.1.3.0) using a two-step database searching approach (27) with the corresponding protein database and the *Homo sapiens* proteome database (downloaded from Uniprot in September 2022) when needed. The false discovery rate was set as 1%. Carbamidomethylation of cysteine was set as a fixed modification. The oxidation of methionine and N-terminal acetylation were set as potential modifications. The maximum missed cleavages of trypsin was set as two. After database searching, 6 samples (A05, B02, E08, E13, G10, and H09) from the LP data set were removed due to the low data quality.

### Statistical analyses

Taxonomic and functional analysis performed by Unipept desktop 2.0 (28) used identified peptide sequences as input. The taxonomic annotation of SIHUMIx data was performed by a custom-targeted protein reference database, which was constructed in the Unipept by manually selecting and combining the information of strains in SIHUMIx. For F01 and LP, the default database was applied. The functional annotation similarity was calculated by MegaGO (29) based on the “biological process” Gene Ontology (GO) annotation provided by Unipept.

We developed a peptide-centric taxonomic annotation approach, termed pepTaxa (Fig. 1C). The output file “peptide.txt” from database searching, in which each identified peptide corresponds to a group of protein IDs, was used as input for the pepTaxa approach. The pepTaxa approach matched the protein ID to taxonomic information in CGR2. The taxonomic annotation for each peptide was determined by calculating the lowest common ancestor (LCA) of taxonomic annotation of the corresponding group of protein IDs.

For the downstream functional annotation of pepTaxa, the function information in CGR2 was used for the functional annotation of the LP data set (Fig. 1C). The KEGG pathway enrichment was analyzed by adapted gene set variation analysis (GSVA) integrated in pepFunk (30). Functional annotation of the results obtained from IGC database searching was performed with the default peptide-to-KEGG database integrated in pepFunk. The results from database searching against DBCGR2 were functionally annotated with a custom peptide-to-KEGG database. To create the custom peptide-to-KEGG database that enables functional annotation in pepFunk, the protein IDs corresponding to one peptide were matched to KEGG orthologs (KOs) according to CGR2. If all KOs in a group were identical, the peptide was annotated with one KO. Conversely, if all KOs in this group were diverse, the peptide was not annotated with any KO.

## RESULTS

### Overview of the workflow

The DBCGR2 database was created based on 3,324 proteomes isolated from the feces of healthy Chinese donors (19, 21) (Fig. 1A). This protein database includes 527 species across 199 genera, 60 families, 33 orders, 12 classes, and 8 phyla. The performances of DBCGR2 were evaluated on three dimensions. The first one was the performance on peptide and protein identification, which is inherently affected by the database choice (Fig. 1B). The database searching results by DBCGR2 were compared with those by matched metagenomics-derived databases or IGC. Furthermore, the proficiency of DBCGR2 in both taxonomic and functional annotation was assessed. To evaluate the performance of DBCGR2 on taxonomic annotation, a novel peptide-centric approach pepTaxa was developed (Fig. 1C) and was subsequently compared with the taxonomic annotation by Unipept (Fig. 1D). The performance of DBCGR2 for functional annotation was assessed by pepFunk combined with a custom peptide-to-KEGG database based on DBCGR2 and was compared with the default built-in peptide-to-KEGG database which is created based on IGC (Fig. 1E).

### Assessment of DBCGR2 performance for metaproteomic database searching

#### Evaluation of DBCGR2 on synthetic microbial community

**Fig 2:**
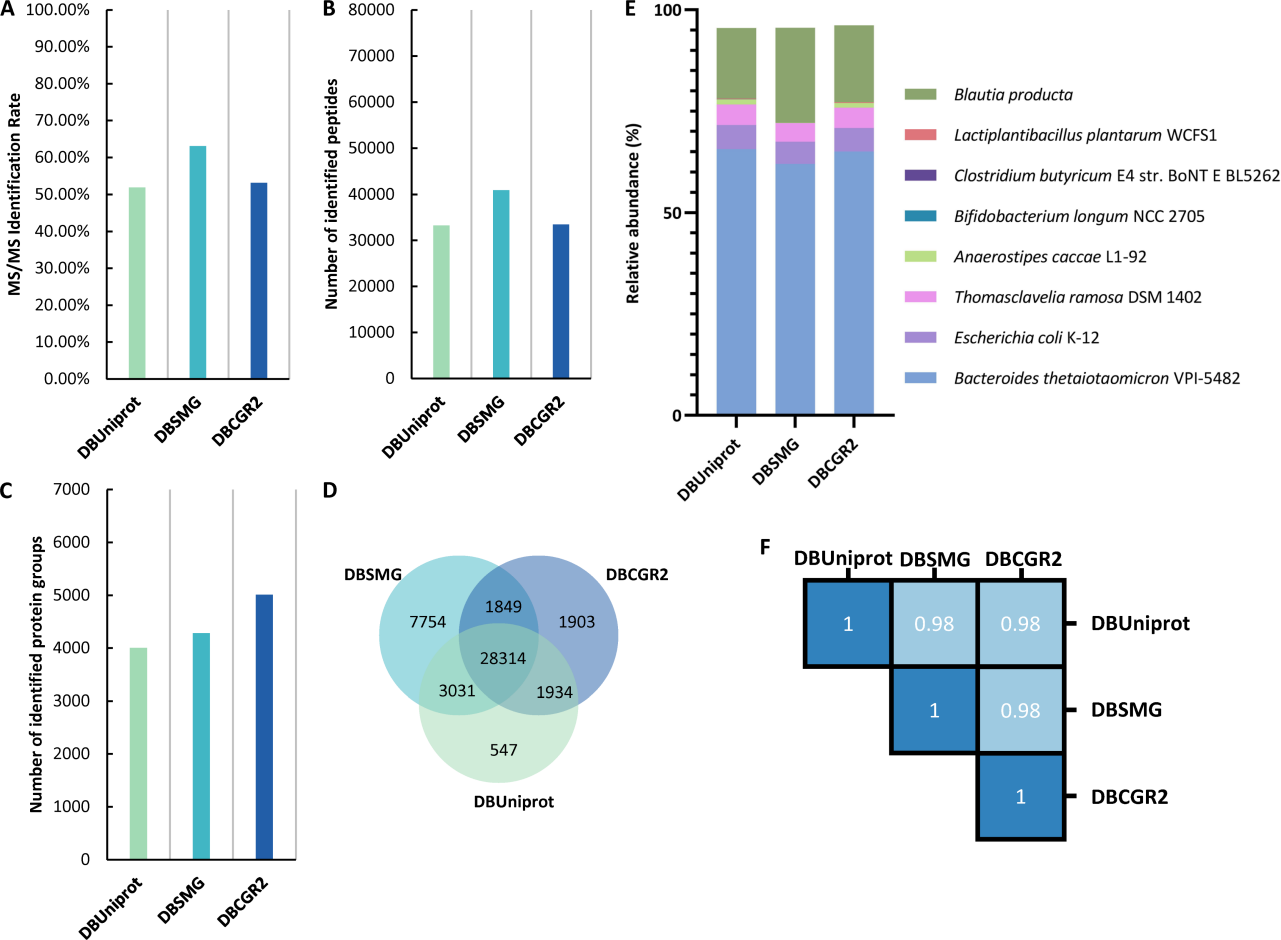
Comparison of the performances of three databases for the SIHUMIx sample S11. The identification results of MS/MS identification rate (**A**) and number of identified peptides (**B**) and protein groups (**C**) by DBUniprot, DBSMG, and DBCGR2. (**D**) Venn diagram of identified peptides. (**E**) Taxonomic composition is calculated based on peptide intensity at the species level. (**F**) Functional similarity of GO domain “biological process” among three databases.

The performance of the DBCGR2 protein database on identification was evaluated on the synthetic gut metaproteomic data set (SIHUMIx) S11, the CAMPI fecal data set F01, and the LP metaproteomic data set. For SIHUMIx S11, the MS/MS identification rate (Fig. 2A) by DBUniprot, DBSMG, and DBCGR2 were 51.94%, 63.14%, and 53.13%, respectively. The numbers of identified peptides (Fig. 2B) were 33,268, 40,930, and 33,460, respectively. The number of identified protein groups (Fig. 2C) was 4,007, 4,285, and 5,013. Over half of the identified peptides were shared by three databases (Fig. 2D), with the highest number of unique identifications attributed to DBSMG.

For the taxonomic annotation (Fig. 2E), the taxonomic patterns quantified by DBUniprot and DBCGR2 exhibited considerable similarity but were different from those of DBSMG. All eight species were identified and quantified using both DBCGR2 and DBUniprot. Peptides of *C. butyricum*, *L. plantarum*, and *B. longum* were not identified by DBSMG. As reported previously, these three strains are not found in the metagenomic database from which the DBSMG was derived (24). Conversely, metaproteomic results from DBUniprot and DBCGR2 showed that the relative abundances of these three strains were lower than 0.2%. Discrepancies were also observed in the relative abundances of *B. producta* and *A. caccae* by DBSMG (Table S1). There were similarities shared by the three databases. *B. thetaiotaomicron* emerged as the most predominant species across all three methods, while *E. coli* maintained approximately 5% abundance. The functional annotation similarity among the three methods was highly similar (Fig. 2F). Collectively, these findings showed the precision of DBCGR2, particularly in the identification of low-abundance strains, demonstrating an advantage over DBSMG.

#### Evaluation of DBCGR2 on fecal samples

**Fig 3:**
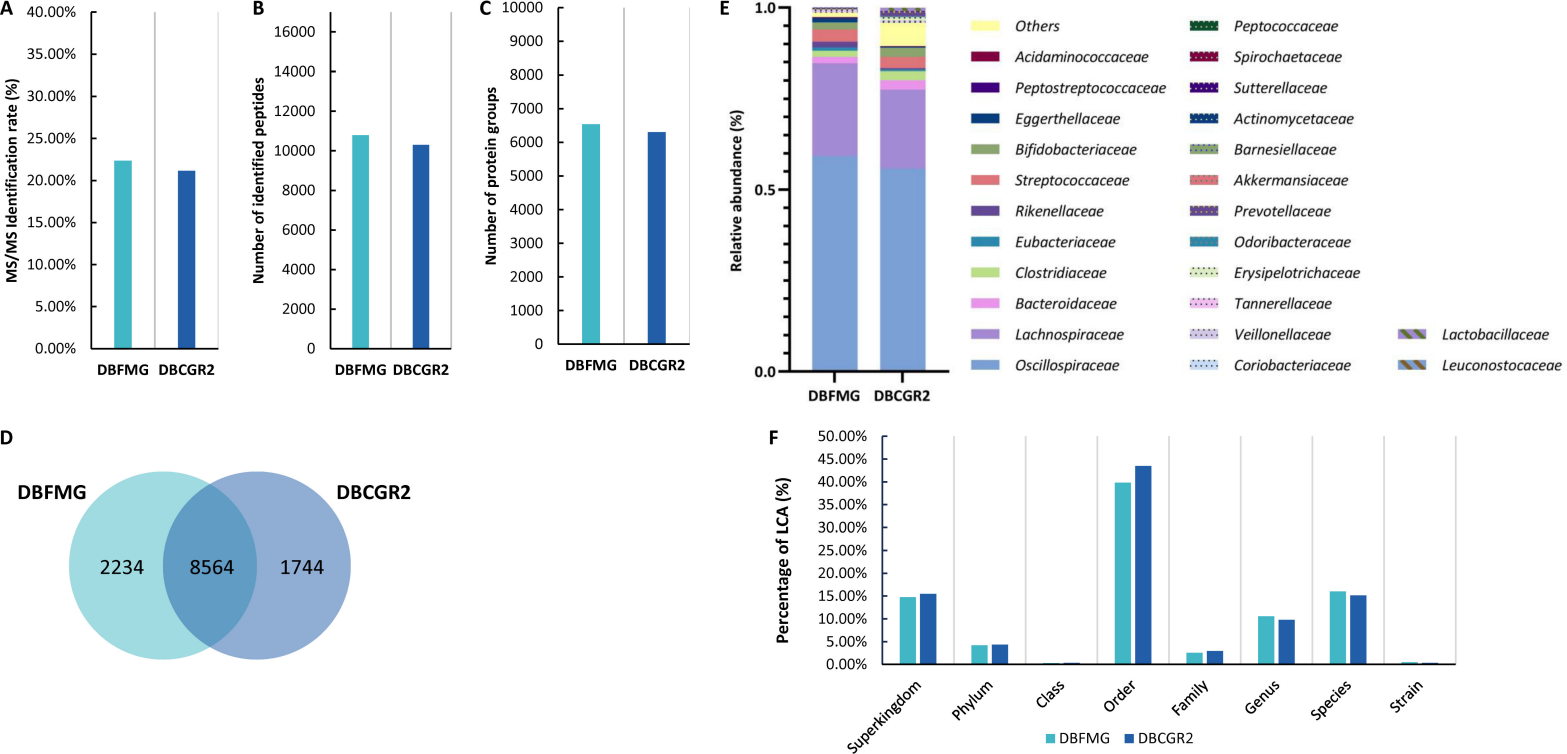
Comparison of the performances of two databases for the CAMPI fecal sample F01. The identification results of MS/MS identification rate (**A**) and number of identified peptides (**B**) and protein groups (**C**) by DBFMG and DBCGR2. (**D**) Venn diagram of identified peptides. (**E**) Taxonomic composition is calculated based on peptide intensity at the family level. (**F**) Distribution of LCA taxonomic ranks.

For CAMPI F01, the MS/MS identification rates using DBFMG and DBCGR2 were 22.36% and 21.15%, respectively (Fig. 3A). We identified 10,396 and 10,798 unique microbial peptides by searching against DBCGR2 and DBFMG, respectively (Fig. 3B). The number of identified protein groups (Fig. 3C) was 6,544 and 6,306. The number of shared peptides was 8,564 (Fig. 3D), with 1,744 peptides uniquely identified by DBCGR2 and 2,234 by DBFMG. Although the MS/MS identification rate, the number of identified peptides, and protein groups were comparably similar, with slightly higher by searching against DBFMG, the peptides identified by DBCGR2 and DBFMG were distinct.

Taxonomic composition based on peptide intensity quantified by DBFMG and DBCGR2 also differed (Fig. 3E), especially for families with less than 2% relative abundance and notably for the “others” category. The results showed that the most abundant two families were Oscillospiraceae and Lachnospiraceae by both databases with slightly different relative abundances. In the “others” quantified by DBCGR2, the top five included Enterobacteriaceae, Bacillaceae, Selenomonadaceae, Enterococcaceae, and Coprobacillaceae (Table S2). In the “others,” DBCGR2 identified 24 families, whereas DBFMG identified 11, indicating an increase in taxonomic diversity with DBCGR2. However, it was noted that the peptides from Eukaryota and Viruses were quantified by DBFMG, while only Bacterial peptides were quantified by DBCGR2 (Fig. S1). The LCA distribution of DBCGR2 and DBFMG database searching results annotated by Unipept revealed highly similar distributions of LCA taxonomic ranks (Fig. 3F), indicating that the taxonomic resolution was dominated by the taxonomic annotation method. The functional similarity of GO annotations between database searching results by DBCGR2 and DBFMG was extremely high (0.96; Fig. 3G), implicating that functional annotations were highly similar across different database searching results.

**Fig 4:**
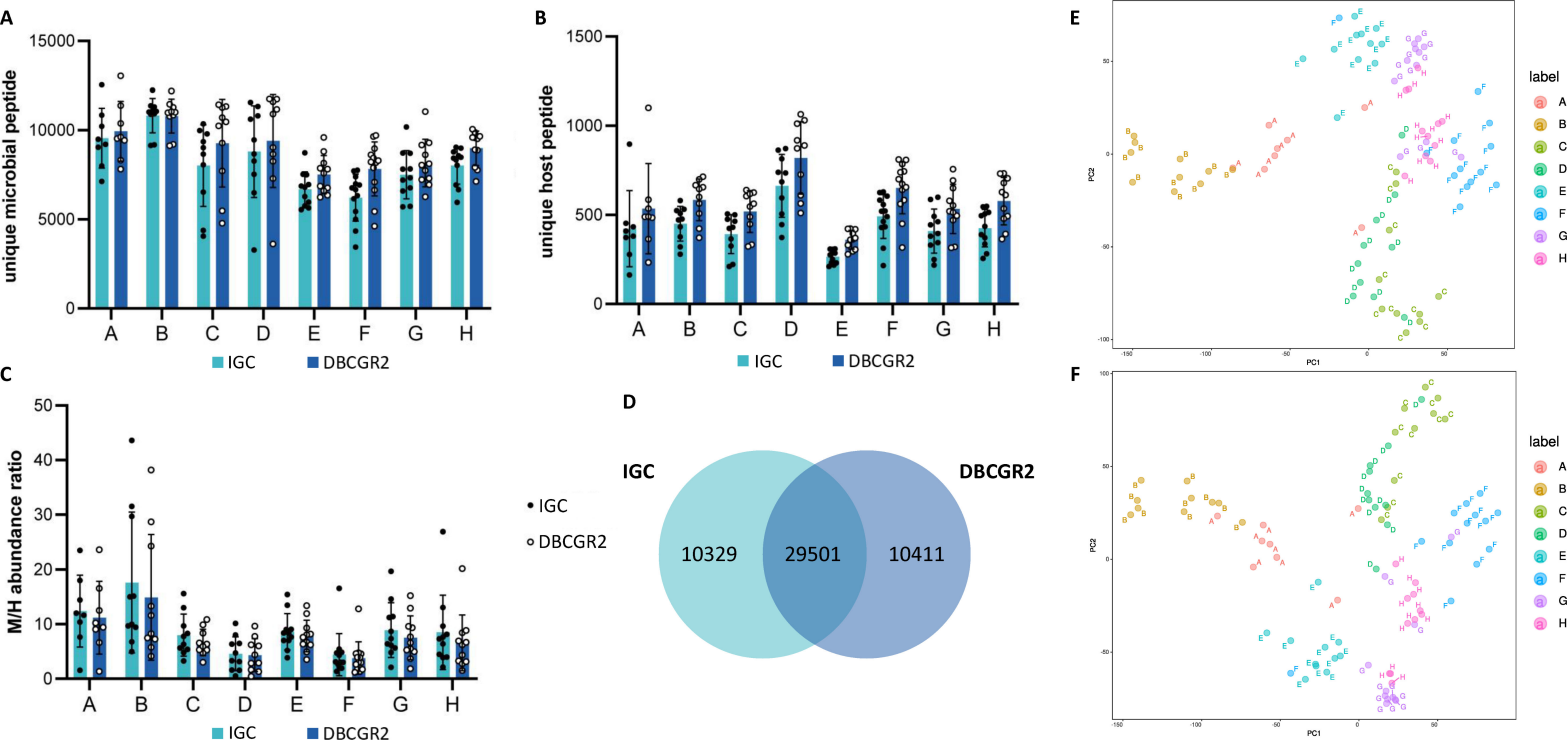
Comparison of the identification rates by IGC and DBCGR2 for the LP data set. The average number of peptides assigned to microbes (**A**) and the host (**B**) of each crew. (**C**) The average intensity ratio of peptides assigned to microbes versus the host of each crew. (**D**) Venn diagram of identified peptides quantified by two databases. Principal component analysis (PCA) plots of peptide abundances quantified by IGC (**E**) and DBCGR2 (**F**). Each dot represented a sample.

For the LP data set (Fig. 4A and B), we identified 39,830 unique microbial peptides and 1,909 unique human peptides using the DBCGR2 protein database for all eight crews. Utilizing the IGC database resulted in the identification of 39,912 unique microbial peptides and 1,476 unique host peptides. The average number of identified unique microbial peptides and unique host peptides using DBCGR2 surpassed those identified by IGC. Notably, the abundance ratios of microbial peptides to host peptides quantified by DBCGR2 were lower than those by IGC (Fig. 4C), attributable to the higher quantity of host peptides by DBCGR2, thereby lowering the M/H ratios. The protein groups that identified in ≥50% samples in each crew member group were 3,574 and 3,584 by DBCGR2 and IGC, respectively.

A comparison of the identified peptides by DBCGR2 and IGC (Fig. 4D) revealed nearly equal numbers of peptides exclusively identified by each database indicating that the composition of identified peptides differed between DBCGR2 and IGC. The more peptides identified by DBCGR2 may be ascribed to the integrity of cultured high-quality genomes, whereas IGC may encompass peptides derived from uncultured organisms. Further comparison between peptides uniquely identified by DBCGR2 and those identified by both IGC and DBCGR2 revealed that peptides from 89 strains (Table S3) were exclusive to DBCGR2, with 27 of these strains belonging to the *Bifidobacterium* genus, corroborating the presence of a large number of high-quality genomes of *B. longum*, *Bifidobacterium pseudocatenulatum*, and *Bifidobacterium adolescentis* in CGR2 (19). This elucidated why peptides identified only by DBCGR2 came from the *Bifidobacterium* genus. The clustering calculated based on peptide intensity was markedly similar between IGC and DBCGR2 database searching results (Fig. 4E and F).

### Evaluation of DBCGR2 for taxonomic and functional annotation

#### Comparison of taxonomic annotation by pepTaxa and Unipept

The performance of DBCGR2 on taxonomic analysis was assessed on the LP metaproteomic data set. The comparison was conducted by three methods. The taxonomic annotation for the IGC database searching result was analyzed by Unipept (method 1), and the taxonomic annotation for the DBCGR2 database searching result was conducted using Unipept (method 2) and pepTaxa (method 3).

**Fig 5:**
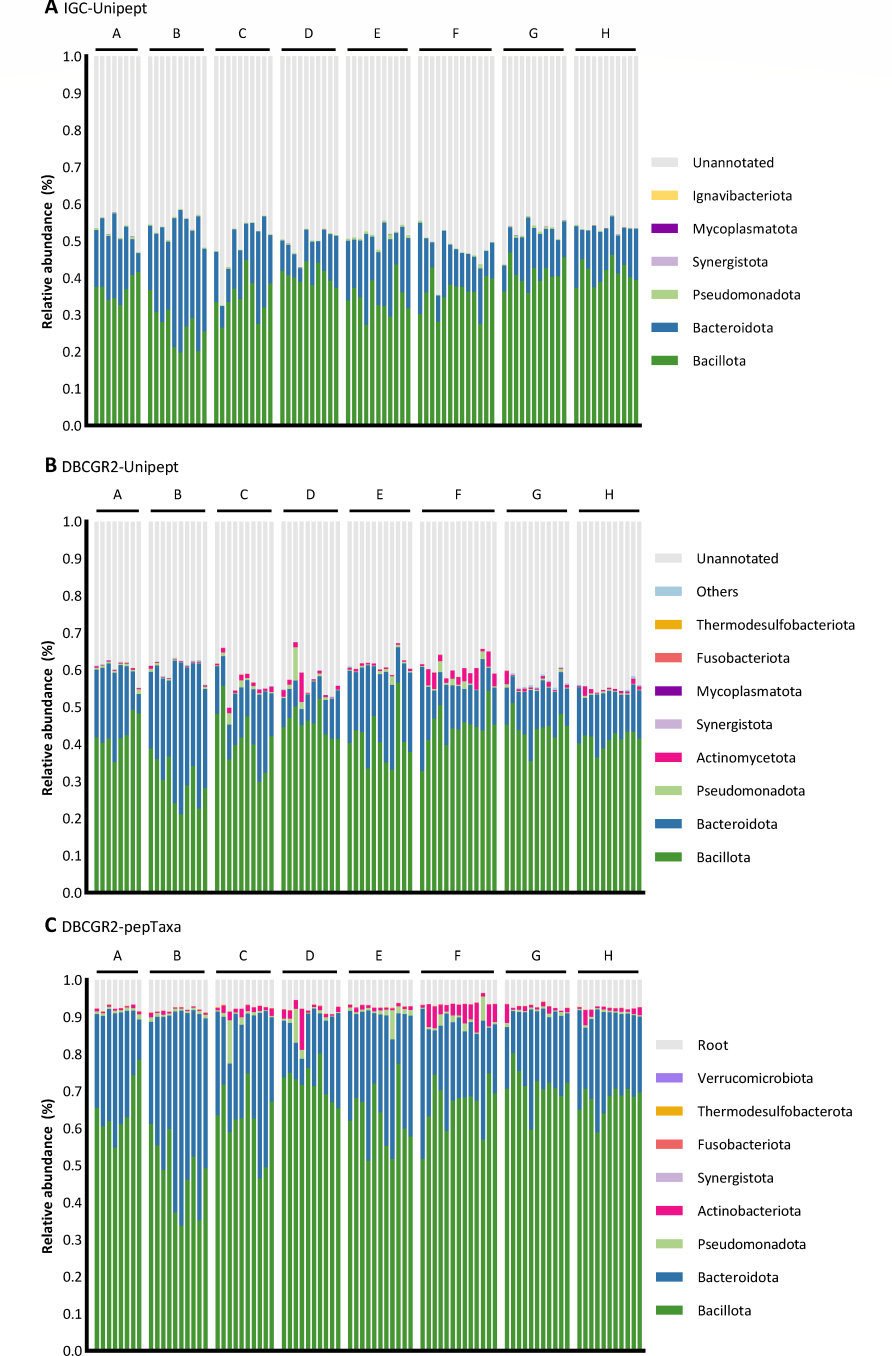
Comparison of taxonomic composition annotated by three taxonomic annotation methods at the phylum level. The taxonomic annotation was performed on all 90 samples. Different taxonomic compositions were annotated by method 1 (**A**), method 2 (**B**), and method 3 (**C**).

**Fig 6:**
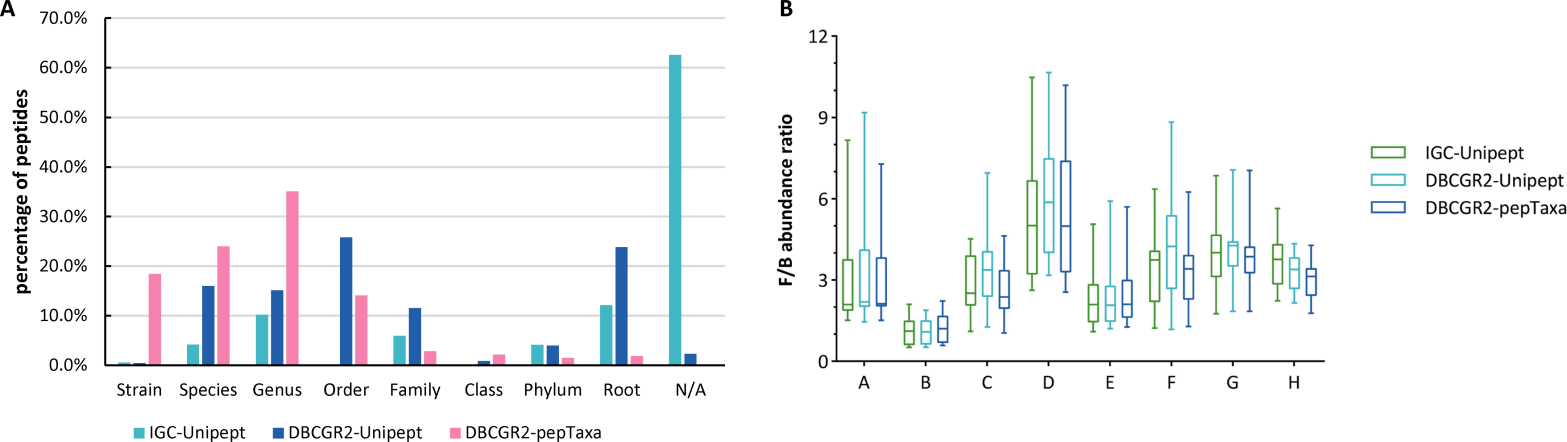
Comparison of three taxonomic annotation methods for the LP data set. (**A**) Distribution of LCA taxonomic ranks calculated based on the number of peptides. Peptides annotated with superkingdom were characterized as “root.” Peptides that cannot be annotated were classified as “N/A.” (**B**) The Firmicutes/Bacteroidetes (Bacillota/Bacteroidota) intensity ratio was calculated based on peptide intensity for each crew.

The relative abundances at the phylum level were determined based on the taxonomic annotations by three methods for each crew (Fig. 5). Taxonomic annotations categorized as “Superkingdom,” “No Rank,” and “N/A” were collectively designated as “Unannotated.” The average relative abundance percentages of “Unannotated” by method 1 for each crew ranged from 46% to 52.2% for each crew, whereas method 2 ranged from 38.5% to 44.7%. Peptides were fully annotated with taxonomy by method 3, with “Superkingdom” reclassified under “Root.” All three methods consistently quantified Bacillota and Bacteroidota as the most dominant phyla. The Firmicutes/Bacteroidetes (Bacillota/Bacteroidota) intensity ratios (Fig. 6B), also a biomarker for obesity (31), calculated based on the peptide abundances quantified by three methods were similar, with deviations for C02, F12, and H03 (Fig. S3). This indicated that the F/B ratios quantified by the three methods were consistent, with variances manifesting in phyla of lower abundance. From Fig. 5, it was observed that Actinomycetota cannot be quantified by method 1, and Mycoplasmatota cannot be quantified by method 3.

The distributions of LCA taxonomic ranks were different across the three methods (Fig. 6A). The most abundant ranks were “N/A,” “Order,” and “Genus” for methods 1–3, respectively. The taxonomic annotation rate was extremely high with 62.6% being “Unannotated” by method 1. The reason for the low annotation rate may be attributed to the considerable presence of uncultured organisms within the IGC, which lack proper taxonomic information. The highest number of annotated peptides was classified under “Family” and “Genus” by method 3, which suggested the high taxonomic resolution by pepTaxa. The category denoted as “Unannotated” by method 2 could be completely annotated through the application of method 3 (Fig. S2A). It is observed from the results of taxonomic annotation that the quantification of composition at the genus level is exclusively achievable by employing method 3, as shown in Fig. S2B. The quantification of genus-level composition is rendered infeasible with methods 1 and 2 due to the observed low rate of taxonomic annotation. The statistical analysis at the species level is even more challenging.

#### Evaluation of DBCGR2 for functional annotation

**Fig 7:**
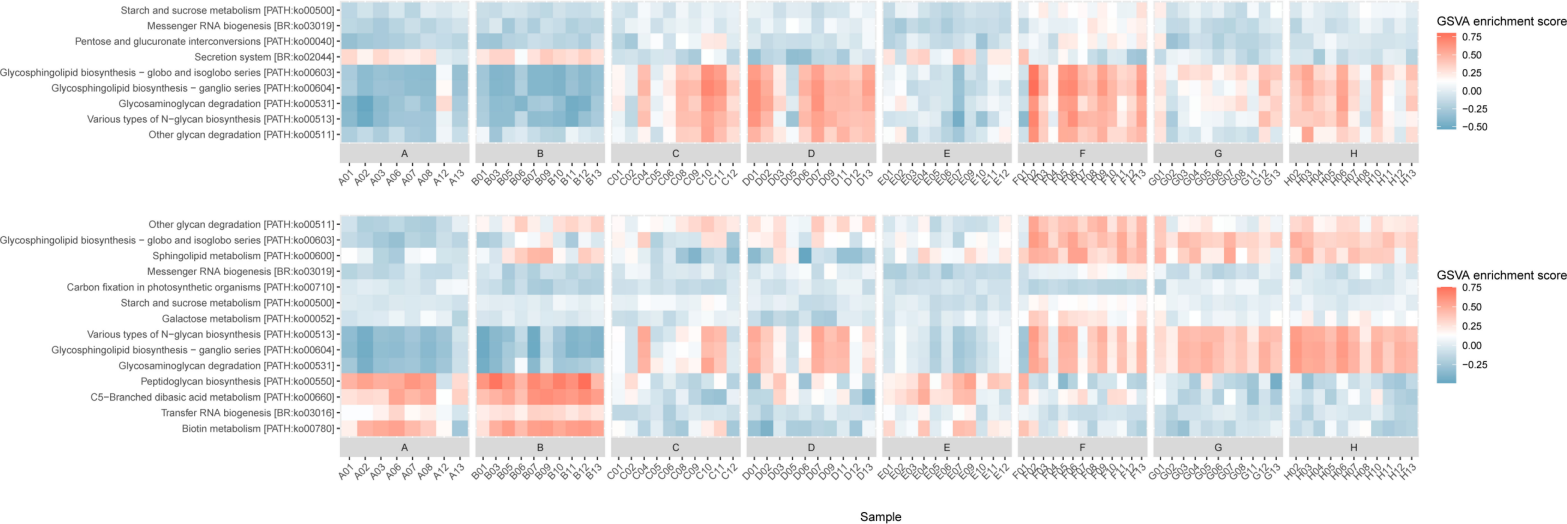
Visualization of significantly enriched KEGG pathway based on GSVA scores of the LP data set. The peptide data were obtained from the results of IGC (top) and DBCGR2 (bottom) database searching. The high GSVA score was colored with coral and the low score with blue. The threshold of the adjusted *P*-value for plotting was *P* < 0.000001.

A significant advantage of metaproteomics is its capacity to identify functionality. In this study, the pepFunk tool was employed to evaluate functional variables among individuals. The results obtained from database searching by DBCGR2 were analyzed using a custom peptide-to-KEGG database created based on DBCGR2. The results from database searching by IGC were analyzed by a default built-in peptide-to-KEGG database. A total of 107 and 117 KEGG pathway peptide sets were processed for GSVA from the results of IGC and DBCGR2, respectively. Analysis revealed 14 significantly enriched KEGG pathways for results from DBCGR2 and nine pathways for IGC (*P*-value < 0.000001), with seven pathways shared by both (Fig. 7). The shared pathways exhibited consistency with minor variations noted in the PATH:ko00513 and PATH:ko00604 in crew G and PATH:ko00603 in crew A, B, C, and D. Variability in functionality was observed across individuals, yet within individuals, functionality remained relatively invariable. Combined, the results showed that DBCGR2 is more sensitive than IGC in functional annotation.

## DISCUSSION

Database construction strategy is vital for metaproteomics research. In this study, the protein database constructed from CGR2 was first utilized in metaproteomic data analysis, and a complete peptide-centric data analysis workflow was demonstrated. The results showed that the performance of DBCGR2 in peptide identification was comparable with matched metagenomics (metatranscriptomics)-derived databases and IGC reference catalog database. For the CAMPI data set S11 and F01, the identification rate by DBCGR2 was marginally lower than the matched metagenomics (metatranscriptomics)-derived databases. From database searching results of the LP data set, despite the IGC database comprising more protein sequences than DBCGR2, the numbers of identified peptides by DBCGR2 and IGC were almost equal. Combined with the identification results from all data sets, it indicated that the size of the protein database is not the only critical factor, which also addresses the importance of the comprehensiveness of the database (10). We also observed that databases derived from sequencing data and cultured genome data resulted in different peptide or protein identification. The DBCGR2 outperformed metagenomics-derived databases in identifying peptides from low-abundance species. For example, the identification of peptides from the Bifidobacterium genus will be useful in studying the function of probiotics in the human gut microbial community. Utilizing DBCGR2 could be time- and cost-saving by eliminating the need to produce matched metagenomics data.

In the taxonomic annotation step, we noted that the taxonomic resolution depended on the annotation method employed. In this study, we developed pepTaxa, a novel peptide-centric taxonomic annotation approach, to enhance the rate and resolution of taxonomic annotation. All peptides identified by DBCGR2 can be fully annotated by pepTaxa, and the taxonomic annotation results demonstrated a better taxonomic resolution. This approach offers a reduction in processing time and expedites the annotation process. Furthermore, it facilitates seamless updates in conjunction with the availability of novel gut microbial genomes in the future.

Metaproteomic data analysis utilizing DBCGR2 and pepTaxa has demonstrated comparable identification capabilities and taxonomic resolution enhancement. This workflow of using DBCGR2 and pepTaxa has limitations. The DBCGR2 was constructed based on CGR2, which has limited bacterial genomes from healthy Chinese adults. Future efforts should incorporate additional Chinese gut microbial genomes, such as those from hGMB (32), particularly for low-abundance taxa, and apply appropriate methods to further improve taxonomic resolution. Its current application is suggested primarily for analyzing metaproteomic data from Chinese adult cohorts due to variations in gut microbiota across different populations, characterized by geographical, age, and ethnic differences. Future efforts will focus on expanding the database with a broader range of cultured genomes to enhance its applicability to diverse cohorts, with subsequent evaluations of its performance on these varied data sets. Moreover, the DBCGR2-pepTaxa workflow should be integrated with functional annotations from other databases, such as carbohydrate-active enzymes (33), for functional analysis demands.

In conclusion, our results suggested that the DBCGR2 derived from culturomics is a feasible alternative in database searching in the processing of metaproteomic data, enabling comprehensive taxonomic annotation of identified and quantified peptides at the same time.

## Data Availability

The DBCGR2, database before removing duplicates, and result files have been deposited to the ProteomeXchange Consortium (34) via the PRIDE (35) partner repository with the dataset identifier PXD053977. The code for taxonomic and functional analysis can be accessed with the dataset identifier PXD053977.

## SUPPLEMENTAL MATERIAL

Supplemental material spectrum.01755-24-s0001.pdf Fig. S1 to S3; Tables S1 to S3.

ASM does not own the copyrights to Supplemental Material that may be linked to, or accessed through, an article. The authors have granted ASM a non-exclusive, world-wide license to publish the Supplemental Material files. Please contact the corresponding author directly for reuse.
