# Supplementary material for: Cultivated genome references for protein database construction and high-resolution taxonomic annotation in metaproteomics
Source: Microbiol Spectr. 2024 Dec 12;13(2):e01755-24. doi: 10.1128/spectrum.01755-24 (PMC11792528; doi:10.1128/spectrum.01755-24)
Supplement: Supplemental material — Fig. S1 to S3; Tables S1 to S3. [file spectrum.01755-24-s0001.pdf]

Supplementary Figures

Figure S1. Taxonomic annotation by Unipept for results identified by DBCGR2 and DBFMG. The figure was calculated based on the number of peptides assigned to different Phyla.

Figure S2. The taxonomic annotation by Method 3. (A) The Sankey plot showed the ‘Unannotated’ categorized by Method 1 can be fully annotated by Method 3. (B) The average relative abundance of top ten genera for each crew.

Figure S3. Firmicutes/Bacteroidetes (Bacillota/Bacteroidota) intensity ratio for each sample.

Figure S1

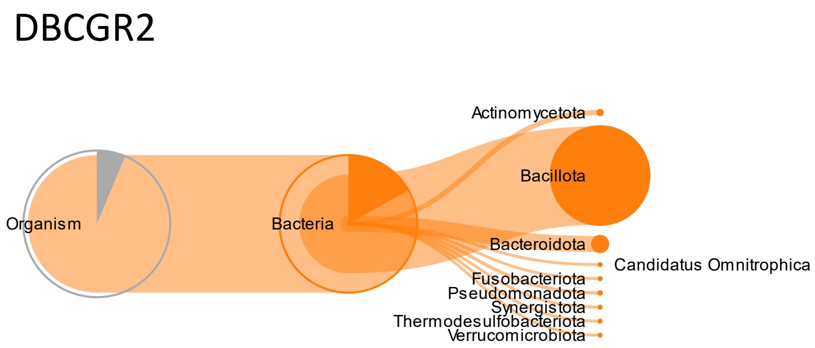
DBCGR2
Actinomycetota
Bacillota
Organism
Bacteria
Bacteroidota
Candidatus Omnitrophica
Fusobacteriota
Pseudomonadota
Synergistota
Thermodesulfobacteriota
Verrucomicrobiota

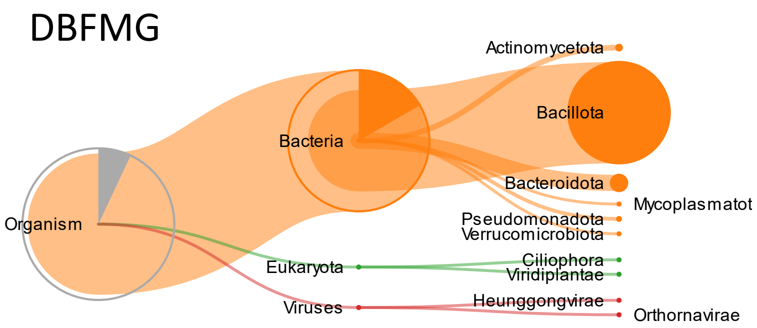
DBFMG
Actinomycetota
Bacillota
Bacteria
Bacteroidota
Mycoplasmatot
Organism
Pseudomonadota
Verrucomicrobiota
Ciliophora
Eukaryota
Viridiplantae
Heunggongvirae
Viruses
Orthornavirae

Figure S2.

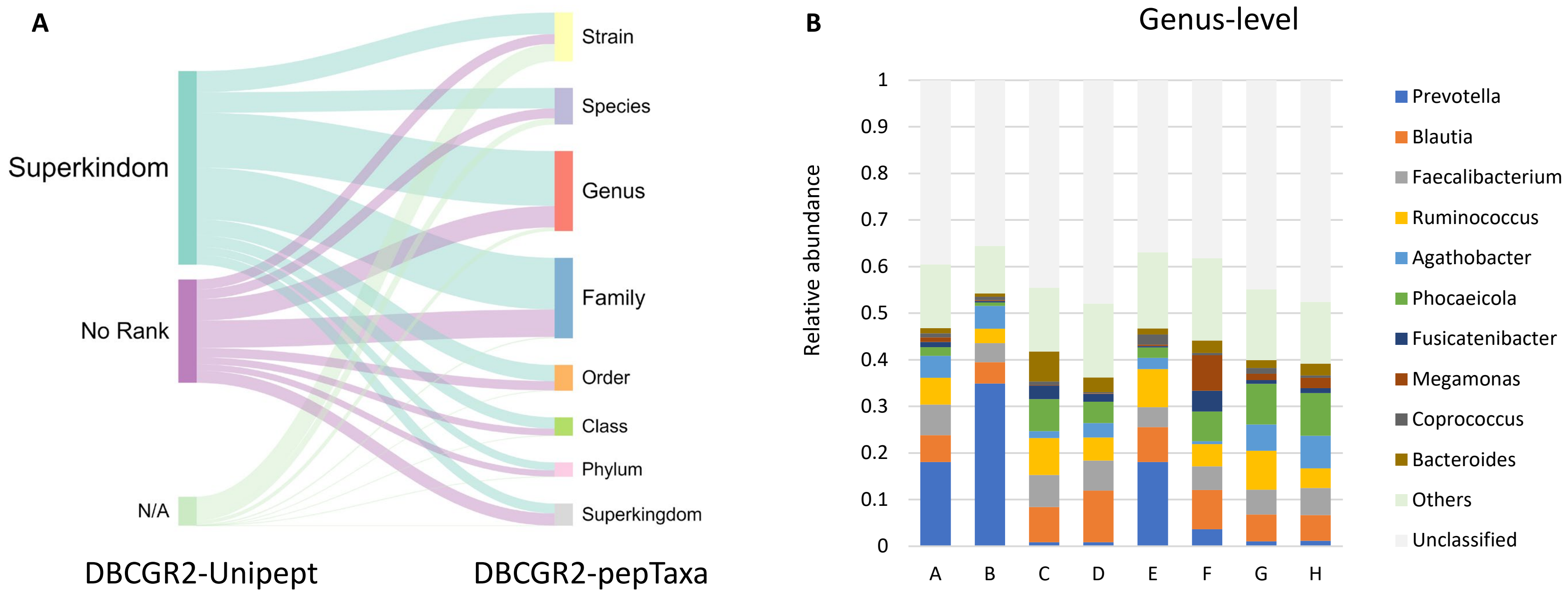
A
Superkindom
No Rank
N/A
Strain
Species
Genus
Family
Order
Class
Phylum
Superkingdom
DBCGR2-Unipept
DBCGR2-pepTaxa
B
Genus-level
Relative abundance
1
0.9
0.8
0.7
0.6
0.5
0.4
0.3
0.2
0.1
0
A
B
C
D
E
F
G
H
Prevotella
Blautia
Faecalibacterium
Ruminococcus
Agathobacter
Phocaeicola
Fusicatenibacter
Megamonas
Coprococcus
Bacteroides
Others
Unclassified

Figure S3.

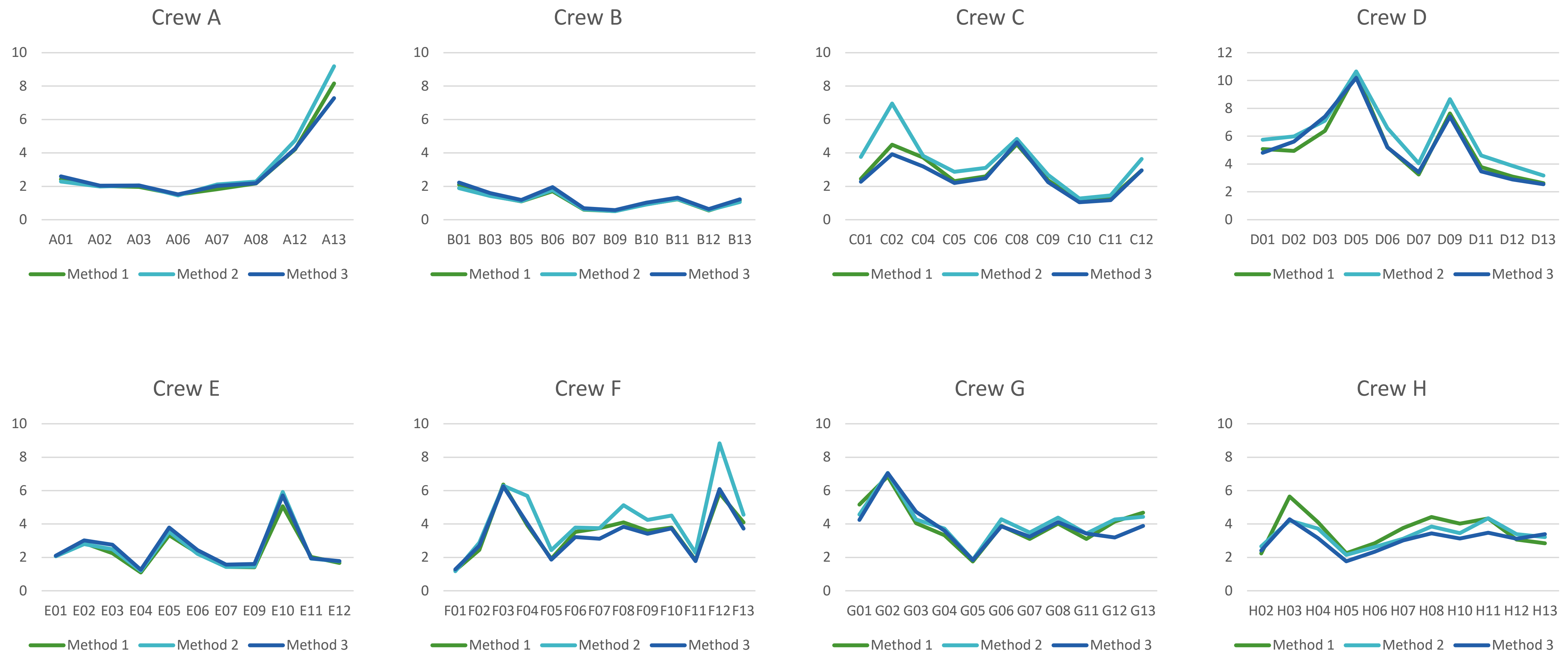
Crew A
10
8
6
4
2
0
A01 A02 A03 A06 A07 A08 A12 A13
Method 1 Method 2 Method 3
Crew B
10
8
6
4
2
0
B01 B03 B05 B06 B07 B09 B10 B11 B12 B13
Method 1 Method 2 Method 3
Crew C
10
8
6
4
2
0
C01 C02 C04 C05 C06 C08 C09 C10 C11 C12
Method 1 Method 2 Method 3
Crew D
12
10
8
6
4
2
0
D01 D02 D03 D05 D06 D07 D09 D11 D12 D13
Method 1 Method 2 Method 3
Crew E
10
8
6
4
2
0
E01 E02 E03 E04 E05 E06 E07 E09 E10 E11 E12
Method 1 Method 2 Method 3
Crew F
10
8
6
4
2
0
F01 F02 F03 F04 F05 F06 F07 F08 F09 F10 F11 F12 F13
Method 1 Method 2 Method 3
Crew G
10
8
6
4
2
0
G01 G02 G03 G04 G05 G06 G07 G08 G11 G12 G13
Method 1 Method 2 Method 3
Crew H
10
8
6
4
2
0
H02 H03 H04 H05 H06 H07 H08 H10 H11 H12 H13
Method 1 Method 2 Method 3

Supplemrntary Tables

Table S1. The comparison of microbial community taxonomic composition of SIHUMIx S11.

	DBUniprot	DBSMG	DBCGR2
Bacteroides thetaiotaomicron VPI-5482	65.60%	62.00%	64.99%
Escherichia coli K-12	5.92%	5.42%	5.82%
Thomasclavelia ramosa DSM 1402	5.12%	4.61%	5.02%
Anaerostipes caccae L1-92	1.24%	0.06%	1.13%
Bifidobacterium longum NCC2705	0.01%		0.01%
Clostridium butyricum E4 str. BoNT E BL5262	0.03%		0.01%
Lactiplantibacillus plantarum WCFS1	0.17%		0.17%
Blautia producta	17.41%	23.48%	19.03%
unknown	4.50%	4.42%	3.83%

Table S2. The comparison of microbial community taxonomic composition of CAMPI fecal sample F01 (Family-level).

Family	Relative abundance DBCGR2	DBFMG
Oscillospiraceae	55.75%	59.32%
Lachnospiraceae	21.68%	25.36%
Bacteroidaceae	2.60%	1.75%
Clostridiaceae	2.54%	1.72%
Eubacteriaceae	0.39%	0.88%
Rikenellaceae	0.36%	1.60%
Streptococcaceae	3.09%	3.35%
Bifidobacteriaceae	2.53%	1.95%
Eggerthellaceae	0.20%	1.16%
Peptostreptococcaceae	0.12%	0.09%
Acidaminococcaceae	0.12%	0.21%
Others	6.39%	1.19%
Coriobacteriaceae	0.27%	0.26%
Veillonellaceae	0.08%	0.07%
Tannerellaceae	0.29%	0.51%
Erysipelotrichaceae	0.95%	0.08%
Odoribacteraceae	0.32%	0.13%
Prevotellaceae	1.22%	0.16%
Akkermansiaceae	0.00%	0.00%
Barnesiellaceae	0.00%	0.00%
Actinomycetaceae		0.04%
Sutterellaceae	0.11%	0.17%
Spirochaetaceae		
Peptococcaceae		
Leuconostocaceae		
Lactobacillaceae	0.96%	

The composition of catogory 'Others'.

Family	Relative abundance DBCGR2	DBFMG
Enterobacteriaceae	23.38%	22.67%
Peptoniphilaceae	2.89%	
Flavobacteriaceae	0.67%	
Azospirillaceae		50.45%
Selenomonadaceae	11.96%	0.00%
Paenibacillaceae	1.75%	0.00%
Nitrobacteraceae		1.99%
Morganellaceae	1.40%	
Coprobacillaceae	10.11%	
Eubacteriales Family XIII. Incertae Sedi	2.08%	
Fusobacteriaceae	2.62%	
Bacillaceae	17.70%	
Desulfovibrionaceae	1.93%	
Dermabacteraceae	0.23%	
Enterococcaceae	11.84%	
Atopobiaceae	0.28%	
Micrococcaceae	1.57%	
Micromonosporaceae		2.09%
Weeksellaceae	0.59%	
Microbacteriaceae	0.25%	

Tetrahymenidae		0.85%
Staphylococcaceae	1.98%	
Carnobacteriaceae	0.04%	0.23%
Methylococcaceae	0.03%	0.17%
Gordoniaceae	0.86%	
Propionibacteriaceae	1.96%	
Synergistaceae	0.61%	
Synergistota	3.26%	
Acetobacteraceae		0.20%
Halieaceae		17.11%
Paracoccaceae		0.89%
Poaceae		3.33%

Table S3. The strain counts that uniquely identified by DBCGR2.

Strain	Count
Amedibacterium intestinale	1
Bacteroides faecis	2
Bacteroides fragilis_A	1
Bacteroides intestinigallinarum	1
Bacteroides ovatus	2
Bacteroides uniformis	2
Bifidobacterium adolescentis	5
Bifidobacterium longum	8
Bifidobacterium pseudocatenulatum	14
Blautia_A massiliensis	1
Citrobacter portucalensis	1
Clostridium_AQ innocuum	1
Clostridium_P perfringens	1
Clostridium_Q fessum	1
Collinsella sp003439125	1
Collinsella sp003462685	1
Collinsella sp900541695	2
Cutibacterium acnes	1
Enterococcus faecalis	2
Enterococcus_B lactis	2
Enterococcus_B thailandicus	1
Enterococcus_C asini	1
Escherichia coli	1
Faecalimonas umbilicata	1
Klebsiella pneumoniae	8
Klebsiella variicola	1
Kocuria marina_A	2
Lacticaseibacillus paracasei	2
Lactobacillus amylovorus	1
Lactococcus garvieae	1
Lactococcus lactis	1
Lactococcus petauri	1
Limosilactobacillus fermentum	2
Limosilactobacillus mucosae	1
Mediterraneibacter lactaris	2
Megamonas funiformis	1
Mesosutterella multiformis	1
NSJ-61 sp003433845	1
Parabacteroides distasonis	2
Phocaeicola dorei	1
Phocaeicola vulgatus	1
Streptococcus infantarius	2
Streptococcus pasteurianus	2
Weissella confusa	2
Total	89
